# Efficacy and safety of warm acupuncture in the treatment of ankylosing spondylitis: A protocol for systematic review and meta-analysis

**DOI:** 10.1097/MD.0000000000024116

**Published:** 2021-01-08

**Authors:** Sha Dang, YuanYuan Ren, BoYi Zhao, XiangWei Meng, Cong Wang, Xin Han, Yang Liu, ChaoYang Zhang

**Affiliations:** a College of Acupuncture-Moxibustion and Tuina, Shaanxi University of Traditional Chinese Medicine, Xianyang; b Xi ’an Hospital of Traditional Chinese Medicine Affiliated to Shaanxi University of Traditional Chinese Medicine, Xi’an, Shaanxi; c The First Clinical Medical College of Shaanxi University of Chinese Medicine, Xianyang China.

**Keywords:** ankylosing spondylitis, protocol, systematic review, warm acupuncture

## Abstract

**Background:**

Ankylosing spondylitis refers to a type of autoimmune disease, which is commonly characterized by joint pain and stiffness, since the disease progression can exhibit joint deformity and other activities limited symptoms. Has significantly impacts on people's work and life. Warm acupuncture as a traditional Chinese therapy, showing several advantages (eg, safety, economy, and less side effects), has been extensively used to treat ankylosing spondylitis. However, its curative effect is supported by limited evidence. Accordingly, the present study aims to comprehensively assess the reliability of warm acupuncture in ankylosing spondylitis treatment.

**Methods:**

Randomized controlled trials were searched from the Chinese Biomedical Literature Database, Chongqing VIP Database for Chinese Technical Periodicals, China National Knowledge Infrastructure, Wanfang, Web of Science, Cochrane Library, PubMed, and EMBASE, regardless of their publication status. The deadline was November 6th, 2020. Two experienced researchers adopted RevMan V.5.3 software for literature selection, data collection, data analysis, and synthesis, respectively. In addition, the quality of the trials involved in this study was measured with the Cochrane Bias risk assessment tool, regardless of language or publication status.

**Results:**

The protocol will be used to assess the efficacy and safety of warm acupuncture in ankylosing spondylitis treatment.

**Conclusion:**

This review reliably evidences whether warm acupuncture is a reliable method for the intervention of ankylosing spondylitis.

**INPLASY registration number:**

INPLASY2020110096.

## 1 Introduction

Ankylosing spondylitis (AS) refers to a chronic inflammatory disease, largely involving the sacroiliac joint, spinal osteophyte, spinal parenchyma, and around the joint. Its early performance includes joint pain, morning stiffness, late joint deformation, limited lumbar movement in all directions, reduced activity of the thoracic vertebra. According to the report, the incidence of AS in China takes up nearly 0.2% to 0.3%.^[1]^ Besides, men are 2 to 3 times more likely to have such a disease than women.^[2–4]^ Moreover, it has been reported that AS patients are more prone to anxiety, depression, and other psychological problems, significantly impacting patients’ life and work.^[5,6]^ More importantly, AS has been suggested to increase the risk of certain diseases (eg, cardiovascular diseases, respiratory diseases, digestive diseases, and reproductive diseases).^[7–11]^ As revealed from recent studies, patients with autoimmune diseases have a higher risk of COVID-19.^[12]^ Thus, the treatment of AS is of high clinical and public health significance.

The existing treatment of AS largely complies with drug therapy. Currently, both the International Society for the Evaluation of Spinal arthritis and the European Rheumatic Alliance recommend nonsteroidal anti-inflammatory drugs (NSAIDs) as first-line agents to clinically treat this disease. In addition, disease-modifying antirheumatic drugs as second-line therapy significantly impacts AS.^[13–15]^ Undoubtedly, these drugs can improve the symptoms of AS and control the progression of the disease.^[16–18]^ However, the adverse reactions brought by this disease should not be ignored. As revealed from existing studies, the use of NSAIDs adversely affects cardiovascular, gastric, and renal function.^[17,19]^ Moreover, NSAIDs also inhibit sperm formation, interfere with sperm motility, and induce sexual dysfunction.^[17]^ The use of biological agents also elevates the risk of infection (eg, hepatitis B and tuberculosis), and biologics are expensive and not affordable for the general population.^[20,21]^ Thus, a safe and inexpensive treatment with no obvious adverse reactions should be found.

Warm acupuncture combines acupuncture and moxibustion. It is elucidated as first inserting the sterilized needle into a certain part of the body with a certain technique, and then placing the moxa stick on the needle handle and subsequently igniting the moxa stick, so the heat will flow into the acupuncture point along the needle body. As a conventional non-drug therapy, it is capable of regulating Yin and Yang, activating blood circulation, and removing blood stasis. Modern studies revealed that warm acupuncture can improve immunity, as well as play an anti-inflammatory and analgesic role by increasing anti-inflammatory cytokine levels and lowering pro-inflammatory cytokine levels.^[22,23]^ Warm acupuncture and moxibustion has been extensively employed in clinic for its safety, low price, and no obvious adverse reactions.^[24]^ However, systematic evaluations have been rarely conducted on the efficacy of warm acupuncture and moxibustion in AS treatment. Thus, this study aims to systematically assess the efficacy and safety of warm acupuncture and moxibustion in AS treatment.

## 2 Methods

### 2.1 Study registration

The registration number is INPLASY2020110096. Abiding by this agreement, this study complies with the Preferred Reporting Items set out in the guidelines for the Systematic Review and Meta-Analysis Protocol statement.^[29]^

### 2.2 Inclusion criteria for study

#### 2.2.1 Type of studies

It will include all randomized controlled trials of warm acupuncture for AS, regardless of language or publication status. To be specific, animal trials, case studies, nonrandomized controlled trials, empirical reports, and reviews will be excluded.

#### 2.2.2 Types of participants

All participants satisfying the diagnostic criteria for AS as revised by the American College of Rheumatology in 1984 will be involved, regardless of race, sex, age, marital status, or educational background.

#### 2.2.3 Types of interventions

The experimental group was only subjected to warm acupuncture treatment. There was no limit on the point selection, needle insertion depth, acupuncture technique, and time of warm acupuncture. For the intervention measures of the control group, drugs, simple acupuncture, moxibustion, fumigation, exercise therapy, massage, and other treatments were included.

#### 2.2.4 Types of outcome measures

##### 2.2.4.1 Primary outcomes

Clinical efficiency, Bath Ankylosing Spondylitis Disease Activity Index, and visual analog scale were the primary outcomes.

##### 2.2.4.2 Secondary outcomes

1. Finger-to-floor distance;
2. occiput to wall distance;
3. Erythrocyte sedimentation rate, C-reactive protein;
4. Adverse reactions.

### 2.3 Search methods

#### 2.3.1 The primary source of data

Randomized controlled trial of warm acupuncture for AS was searched till November 6th, 2020 from the Chinese Biomedical Literature Database, Chongqing VIP Database for Chinese Technical Periodicals, China National Knowledge Infrastructure, Wanfang, Web of Science, Cochrane Library, PubMed, and EMBASE. The retrieval strategies adopted by PubMed are elucidated in Table 1.

**Table 1:** Search strategy for PubMed.

Number	Search items
#1	randomized controlled trial [pt]
#2	controlled clinical trial [pt]
#3	randomized [tiab]
#4	clinical trials as topic [mesh: noexp]
#5	randomly [tiab]
#6	trial [ti]
#7	OR/#1–#6
#8	animals [mh] NOT humans [mh]
#9	#7 NOT #8
#10	Ankylosing Spondylitis[Mesh]
#11	Ankylosing spondylitis[All Fields)
#12	Spondyloarthritis[All Fields)
#13	Spondyloarthropathies[All Fields)
#14	Seronegative Spondyloarthropathies[All Fields)
#15	OR/#10–#14
#16	Warming acupuncture[Mesh]
#17	Warming acupuncture Therapy[All Fields)
#18	Needle warming moxibustion[All Fields)
#19	OR/#16–#18
#20	#9 AND #15 AND #19

#### 2.3.2 Search of other resources

Some unfinished or unpublished experimental data were retrieved from the Chinese Clinical Trial Registry and The Clinicaltrials.gov.

### 2.4 Data collection and analysis

#### 2.4.1 Literature selection

**Figure 1:**
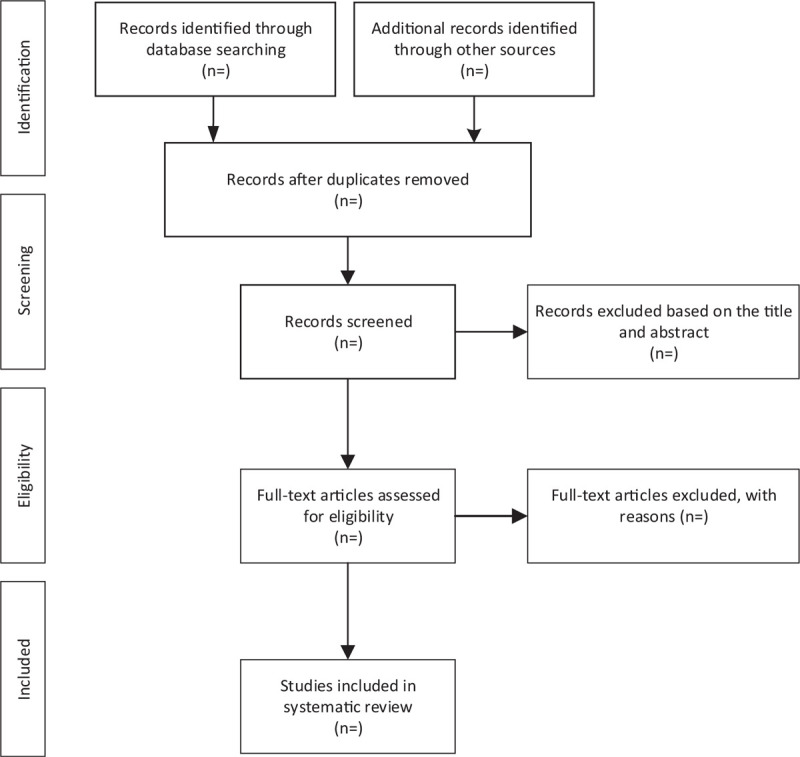
Flow diagram of study selection process.

First, all the literature was imported into the EndNote X9 software and all duplicate literature was deleted. Second, SQ and YX were adopted to review the titles and abstracts, and the irrelevant literature was removed. Third, the full text should be read to determine if the project will be included here. Lastly, 2 researchers (SQ and YX) carried out the cross-check. If there was any disagreement, the third researcher (LJ) would participate in the discussion and solve it. Figure 1 illustrates a flow chart of literature screening.

#### 2.4.2 Data extraction and management

Two researchers will each extract the qualified data into a pre-made table, and a third will step in to resolve any potential differences. The extracted data consisted of journal, author information, title, publication date, participant characteristics, sample size, interventions, study methods, primary and secondary outcome measures, as well as any adverse events.

### 2.5 Risk of bias assessment

SQ and YX employed the Cochrane Bias risk Assessment tool to determine the quality of the trials, respectively.^[29]^ The extracted details were as follows: the random sequence generation, the blindness of result evaluation, the blindness of participants and personnel, the concealment of allocation, the reporting of selective results, the incomplete result data, and so on. These fell to 3 levels, that is, fuzzy, low, and high. In case of ambiguity, the author of the relevant project would be contacted. If there was any dispute, an informed decision would be made with the assistance of the Third investigator (LJ).

### 2.6 Data synthesis

Meta-analysis was conducted with ReviewMan 5.3 software provided by Cochrane, and bivariate data were expressed as risk ratio and 95% confidence interval. Data of continuous variables were expressed by standardized mean difference and 95% confidence interval. The heterogeneity between the results by conducting Chi-square test analysis (alpha test level = 0.1), and combining with quantitative judgment of whether *I*^2^ heterogeneity is big or not. if *I*^2^ ≤ 50%, *P* ≥ .1, it shows good homogeneity between the various research, the fixed effects model would be used; if not, the statistical heterogeneity between the results of the study would be larger, sources of heterogeneity should be studied in depth, and the random effects model would be used. If the level of clinical heterogeneity was significant, sensitivity analysis would be conducted; otherwise, only descriptive analysis would be conducted.

### 2.7 Management of missing data

If the data used in the study was not clear or available, the author of the article would be contacted by telephone or E-mail to get as complete a data set as possible. If the fetch failed, only existing data would be used for analysis.

### 2.8 Subgroup analysis

If there were significant heterogeneity between the trials involved, the course and sample size, type, time, and frequency of warm acupuncture would be considered for subgroup analysis.

### 2.9 Sensitivity analysis

Sensitivity analysis was conducted to exclude low-quality literature to ensure the stability and accuracy of the conclusions drawn from this meta-analysis.

### 2.10 Assessment of reporting biases

If the number of RCTS exceeded 10, funnel plot analysis would be required to test for publication bias. In addition, if there was an asymmetric funnel graph, the Egger check would be conducted to study the causes of publication bias.

### 2.11 Quality of evidence

Two researchers were present to measure the evidence quality of outcome indicators with Grade profiler 3.6 software, with the evaluation scale involving high grade evidence, intermediate evidence, low evidence, and very low evidence.^[30]^

### 2.12 Ethics and dissemination

This study is not related to the patient's personal information, so no ethical approval is required. The results are expected to be published in peer-reviewed journals.

## 3 Discussion

AS refers to an autoimmune disease, early performance for joint pain and morning stiffness, late will appear joint deformation and other symptoms, to the patient and his family brought a heavy burden.^[6,7]^ Drug therapy is currently considered the main effective treatment for AS,^[16,25,26]^ whereas it can cause various side effects; some drugs are expensive, which cannot be afforded generally.^[17,20–22,27]^ As a traditional therapy, warm acupuncture and moxibustion has been extensively used in clinical practice for its advantages (eg, safety, no obvious adverse reactions, and low price). Moreover, several studies reported that acupuncture and moxibustion can regulate the level of inflammatory factors in the body, as an attempt to improve the related symptoms of the human body.^[23,24,28]^ However, as far as the current study is concerned, the efficacy and safety of warm acupuncture and moxibustion in AS treatment are not supported by data. Thus, this study is expected to evidence the clinical use of warm acupuncture and moxibustion in AS treatment.

However, this study may have some potential weaknesses. First, this study did not limit the language types, which may to some extent impact the results. Second, the reliability of this review is related to the methodological quality and comprehensiveness of the studies involved in this program.

## Author contributions

**Conceptualization:** Sha Dang, YuanYuan Ren.

**Data curation:** Sha Dang, Chao Yang Zhang.

**Formal analysis:** Sha Dang, ChaoYang Zhang, YuanYuan Ren.

**Funding acquisition:** YuanYuan Ren.

**Investigation:** Xiang Wei Meng, Xin Han.

**Methodology:** Sha Dang, YuanYuan Ren.

**Resources:** ChaoYang Zhang.

**Software:** Sha Dang, BoYi Zhao.

**Supervision:** Cong Wang, YuanYuan Ren.

**Validation:** Yang Liu.

**Writing – original draft:** Sha Dang, YuanYuan Ren.

**Writing – review & editing:** Sha Dang, YuanYuan Ren.
